# Policy integration of pediatric oral health: aligning medicine and dentistry for child well-being

**DOI:** 10.3389/froh.2026.1716091

**Published:** 2026-03-05

**Authors:** Laresh N. Mistry, Saudamini More, Sumeet Agarwal, Prasad Mhaske, Vivek Sharma, Shreyas Neelkanthan

**Affiliations:** 1 Department of Pediatric and Preventive Dentistry, Bharati Vidyapeeth (Deemed to be University) Dental College and Hospital, Navi Mumbai, India; 2 Department of Public Health Dentistry, Bharati Vidyapeeth (Deemed to be University) Dental College and Hospital, Navi Mumbai, India; 3 Department of Prosthodontics, Bharati Vidyapeeth (Deemed to be University) Dental College and Hospital, Navi Mumbai, India; 4 Department of Periodontology, Bharati Vidyapeeth (Deemed to be University) Dental College and Hospital, Navi Mumbai, India

**Keywords:** dentistry, health equity, health policy, interprofessional relations, oral health, patient centered care, pediatric dentistry, preventive health services

## Abstract

Oral health remains a critical yet overlooked aspect of pediatric health, despite strong evidence linking untreated dental disease with systemic complications, impaired growth, and reduced quality of life. Globally, early childhood caries and related oral conditions affect 60%–90% of children, disproportionately impacting disadvantaged groups. However, pediatric oral health is often siloed from mainstream child health services. Pediatricians, frequently the first point of contact for families, have limited oral health training, while pediatric dentists often see children only when conditions have advanced, reducing opportunities for prevention and early care. Structural barriers—including fragmented professional education, poor referral systems, inadequate insurance, and inconsistent access to preventive services—further limit progress. Families face significant out-of-pocket costs, particularly in low- and middle-income countries, where preventive oral care is excluded from many pediatric health packages. Caregiver health literacy and cultural beliefs also contribute to delayed care-seeking. A policy-level response is essential to bridge these gaps. Key strategies include integrating oral health into routine pediatric visits, advancing interprofessional education, expanding insurance coverage for preventive services, and adopting innovative care models such as telehealth and task-shifting. Embedding pediatric oral health into universal health coverage and child health policies will promote equity and sustainability. Urgent systemic reforms are needed to position pediatric oral health as a core component of child well-being, requiring coordinated action among pediatricians, pediatric dentists, and public health policymakers.

## Introduction

Dental diseases remain among the most pervasive and consequential non communicable conditions worldwide. Their health, economic, and social impacts fall disproportionately on children living in socioeconomically disadvantaged communities, where preventable oral diseases perpetuate inequity across the life course. Contemporary global analyses underscore that poor oral health is not a narrow clinical issue confined to dentistry but a fundamental public-health challenge shaped by social and commercial determinants and by systemic deficits embedded within health systems (1).

Early Childhood Caries (ECC) epitomizes this global burden. Recent meta-analyses estimate that roughly half of preschool-aged children worldwide exhibit some evidence of ECC, with striking geographic variation and a consistent concentration among children facing socioeconomic adversity (2). Once established, ECC often progresses rapidly, leading to pain, sleep disruption, difficulty eating, impaired nutrition, slowed growth, and measurable declines in oral health–related quality of life. These consequences, in turn, may influence children's behavioral, cognitive, and developmental trajectories (2, 3).

Pediatric clinicians, pediatricians and family physicians, serve as the earliest and most trusted points of contact for families, uniquely positioning them to deliver anticipatory guidance, conduct early oral-health risk assessments, apply preventive fluoride interventions, and coordinate timely referral to dental providers. Evidence synthesized by the United States Preventive Services Task Force (USPSTF) affirms the effectiveness of topical fluoride in preventing caries among young children at heightened risk. However, routine oral health examinations in primary care have not demonstrated clear benefits in improving pediatric oral health outcomes because current evidence is insufficient to assess the balance of benefits and harms of routine screening and preventive interventions performed by primary care clinicians in children and adolescents (4).

Despite clear opportunities, integration of oral-health prevention into routine pediatric practice has been inconsistent. Scoping reviews reveal that pediatric medical trainees receive limited structured education in oral health, contributing to uneven knowledge, variable clinical confidence, and inconsistent referral patterns. Communication pathways between medical and dental providers remain fragmented, resulting in disjointed care for children who are most vulnerable (5). Evaluations of care models similarly demonstrate numerous local innovations but limited scalability or sustainability, perpetuating persistent gaps in integration between pediatric medicine and pediatric dentistry (6).

Importantly, this fragmentation is not merely theoretical. Emerging evidence from pragmatic, multilevel intervention trials shows that when pediatric primary-care teams receive structured training, system-level supports, and integrated workflows, measurable improvements follow. Such interventions increase preventive dental visits and decrease the prevalence of untreated dental disease among children receiving care in primary-care settings, demonstrating both feasibility and public-health significance (7).

Taken together, these findings reveal a deeply entrenched, multilevel challenge, spanning patient factors, clinical training, and structural health-system design. Meaningful progress in pediatric oral health will therefore require coordinated educational reforms, integrated clinical pathways, and supportive policy frameworks that bridge the longstanding divide between pediatric medicine and pediatric dentistry. Only through such comprehensive, system wide approaches can the global burden of ECC and related oral diseases be reduced equitably and sustainably.

## Problem statement

Dental caries is among the most prevalent non-communicable diseases (NCDs) globally. According to WHO (8), nearly half of the world's population, about 3.5 billion people, suffer from oral diseases across the life course, with untreated dental caries representing a major component of this burden. ECC contributes substantially to this global oral-disease load. In India, a recent systematic review and meta-analysis reported a pooled ECC prevalence of approximately 46.9% among preschool children (9). Despite this burden, early oral-health services remain markedly underutilized. Infants often have frequent contact with medical providers through routine well-child visits during the first year of life; however, although professional guidelines recommend establishing a dental home and completing the first dental visit by 12 months of age, cohort analyses of Medicaid-enrolled children show that fewer than 1% receive a dental visit during the first year of life (10, 11). Although dental utilization increases gradually with age, U.S. national survey data show that only about half of children aged 1–4 years receive any dental examination or cleaning each year (12).

Pediatricians are uniquely positioned to deliver early preventive guidance, yet numerous studies document substantial gaps in oral-health knowledge, including limited familiarity with fluoride recommendations and early-childhood preventive guidelines, with the magnitude of deficits varying by region and survey instrument (13, 14). As a result, oral disease often remains undetected until it progresses, leading to symptomatic, complex, and costly care. System-level barriers, such as limited integration between medical and dental services, insufficient provider training, and inadequate dental networks, further compound these challenges (15). Collectively, these factors reflect structural shortcomings in early childhood healthcare delivery, underscoring the need for integrated, prevention-focused models to reduce ECC and improve early oral-health outcomes.

## Oral–systemic connections and implications for child health

Pediatric oral diseases, particularly early childhood caries (ECC), are increasingly recognized as biologically and socially intertwined with broader child health and well-being rather than isolated conditions of the dentition (16, 17). Chronic oral infection and inflammation can influence systemic pathways through immune activation, inflammatory mediators, and microbial translocation, with potential implications for growth, nutrition, sleep quality, and overall health status (16). Conversely, systemic conditions and social determinants—such as poor nutrition, psychosocial stress, and limited access to care—can exacerbate oral disease risk, reinforcing a bidirectional relationship between oral and general health (17).

Conceptual frameworks integrating biological, behavioral, and social pathways describe pediatric oral disease as the cumulative result of influences spanning from “cells to society,” highlighting how early-life oral conditions reflect broader systemic and environmental exposures (16). From this perspective, the persistent separation between pediatric medical and dental services may constrain opportunities for early risk identification, coordinated prevention, and holistic management. Recognizing oral health as an integral component of child health therefore provides a biological and public health rationale for closer integration between pediatric primary care and dental services (17).

## Scattered professional development: inadequacies of formal education

A major contributor to fragmented pediatric oral health care is the lack of coordinated, cross-disciplinary formal education. Pediatricians receive minimal structured oral-health training during undergraduate medical education and residency. Although oral-health content is appearing more frequently in pediatric programs, its depth and consistency vary widely; many curricula rely on short modules rather than longitudinal integration. Evidence shows that structured approaches produce more meaningful improvements. A pre–post evaluation demonstrated that pediatric medicine residents and nurse practitioner students experienced statistically significant improvements in oral-health knowledge, confidence, and clinical skills following a structured curriculum (*p* < .001) (18).

However, brief educational exposures even when effective in the short term may not support long-term behavioral change or consistent clinical practice. Sustained curricular integration, Continuing Professional Development, related knowledge and structured clinical exposure are necessary to promote durable improvements in oral-health competence. As a result of inconsistent training, many pediatricians continue to report limited confidence in performing oral examinations, detecting early signs of caries, and providing evidence-based anticipatory guidance on oral hygiene, fluoride, and diet.

Conversely, pediatric dentists receive extensive didactic and clinical preparation in caries prevention and management but often receive limited structured education on systemic pediatric medicine, development, and chronic health conditions influencing oral outcomes. Supporting this multidisciplinary perspective, Moynihan and Kelly (19) provide robust evidence linking free-sugar intake to caries development, underscoring the importance of shared preventive responsibilities between medical and dental professionals.

Educational isolation limits the development of shared competencies and coordinated care pathways. Earlier analyses highlighted the systemic and economic consequences of fragmented pediatric oral health care (20), and more recent global reviews reinforce these findings. Notably (1), observed that fragmented health-professional education continues to impede the prevention and early identification of dental caries worldwide, emphasizing the need for integrated professional training models.

Interprofessional education (IPE) has demonstrated clear benefits in bridging these gaps. Silk et al. (21) found that a structured medical–dental IPE program significantly improved pediatric residents’ and dental trainees’ knowledge, confidence, and self-reported practice in caries risk assessment, anticipatory guidance, and referral for pediatric oral health. Additional evidence from Czarnecki et al. (22) shows that integrated IPE activities enhance collaborative self-efficacy and improve readiness for coordinated care among both medical and dental learners. Without IPE, pediatric oral health care continues to be delivered in isolation, often not comprehensive oral systemic care, resulting in inconsistent referrals, delayed preventive interventions, and missed opportunities during routine pediatric visits. Embedding comprehensive oral-health content and IPE competencies throughout health-professional curricula is essential for improving prevention, early identification, and timely management of pediatric oral disease.

## The collaboration gap in practice

The “collaboration gap” in pediatric oral health refers to the persistent disconnect between pediatric medical care and pediatric dental care, which leads to delayed detection, limited preventive interventions, and avoidable progression of ECC. Recent evidence illustrates that although primary care physicians frequently see infants and young children during the age period when ECC first develops, they often lack adequate oral-health training, confidence, or structured pathways to provide preventive counselling or timely referral.

Survey-based findings show that general practitioners and pediatricians generally perform basic oral examinations but score poorly on knowledge of caries prevention and management, and few have received any formal training in pediatric oral health (23). This contributes to inconsistent preventive practices and underutilization of anticipatory guidance during routine well-child visits.

The downstream effects of this gap are evident in patterns of late referral to dental services. Children with severe- Early Childhood Caries (s-ECC) frequently present to pediatric dentists only after disease has advanced to the point where extensive treatment—often including general anesthesia (GA) is required. A recent study of Thai children with s-ECC found that those treated under GA had significantly higher caries burden, greater treatment complexity, and poorer oral health indicators compared with children managed through behavior-based, non-pharmacological approaches, reflecting late and reactive referral patterns (24). This doesn't directly suggest delayed referrals but rather points to the fact that s-ECC has increased burden of caries which can be better managed preventively if early referrals are made.

Similarly, a systematic review demonstrated that while GA-based full-mouth rehabilitation temporarily resolves active disease, relapse rates remain high when preventive follow-up is not integrated within medical and dental care systems. Across included studies, caries recurrence after GA ranged from 24% to 59%, and repeat GA occurred in up to 31.8% of cases, highlighting that late-stage intervention alone cannot compensate for the absence of early preventive care and weak interprofessional collaboration (25).

The absence of structured communication strategies between pediatricians and pediatric dentists also creates growing gaps in oral health concerns. The pediatric health care profession has been unable to establish electronic health record connectivity for referrals, cross-institutional referral pathways, or collaborative care pathways that allow pediatricians in particular to receive advice from pediatric dentists once the child has visited in person. Interventions that increase earlier and routine care, particularly around prevention with anticipatory guidance, seem to elude this care gap. This sort of silo approach is uncoordinated; the child could repeatedly access care with health professionals, only to remain undiagnosed or with a lack of preventive intervention timing.

The main claims discussed, categorizing them by evidence strength and by whether they arise predominantly in public or private healthcare contexts and key recommendations can be seen in Table 1.

**Table 1:** Evidence strength for claims related to medical–dental integration in early childhood oral health.

Claim	Evidence strength	Sectoral relevance	Key recommendations
Limited pediatrician/GP knowledge and practice in children's oral health	Strong	Public & Private	Oral-health modules in pediatric/GP training; mandatory CPD; oral-health checklists in well-child visits
ECC frequently undetected until advanced disease requiring GA	Moderate	Public & Private	Early dental referral (first tooth/12 months); medical risk-assessment tools; referral-tracking systems
High relapse, repeat GA, or new caries after GA treatment	Strong	Public & Private	Mandatory post-GA recall (3-monthly); caregiver counselling; fluoride varnish; structured GA follow-up pathways
Fragmented referral pathways and lack of preventive follow-up	Moderate	Predominantly Public	Audit referral systems; shared EHR alerts; integrated or co-located medical–dental care models
Early preventive intervention reduces severe ECC and GA need	Strong	Public & Private	Anticipatory guidance in infancy; dental home by age 1; physician-applied fluoride varnish programs

## Policy and systemic barriers

Despite growing recognition of oral health as a component of overall child well-being, many countries still lack comprehensive and prevention-oriented pediatric oral-health policies. Recent global assessments demonstrate that oral health remains insufficiently integrated into child-health strategies, particularly in low- and middle-income countries, where national oral-health programs when available tend to emphasize treatment rather than prevention (8).

The 2022 WHO Global Oral Health Status Report further highlights that large segments of the global population lack access to preventive care, and most countries have not operationalized oral health within universal health coverage frameworks (8).

A major systemic barrier arises from fragmented intersectoral collaboration. Although school-based oral-health programs exist in many settings, they are poorly connected to primary care systems, resulting in weak referral pathways and inconsistent follow-up. A 2020 systematic review of school-based oral-health programs found that while these initiatives can improve oral-health knowledge and short-term behaviors, they often fail to achieve sustainable reductions in caries when they operate without integration into primary health services or community-level prevention systems (26).

Workforce shortages further exacerbate inequities. The WHO's most recent oral-health workforce analyses identify severe maldistribution of dental providers, with rural and underserved communities experiencing the lowest workforce density. Many countries report 3.38 global average dentists per 10,000 population (highest in Americas 5.67 per 10,000 to lowest in Africas 0.33per 10,000), reflecting shortages in both dental personnel and pediatric specialists (8). This imbalance limits early diagnosis, continuity of care, and timely management of ECC —conditions highly sensitive to delayed intervention.

Financing and coverage policies also function as major barriers. A 2020 global commentary on childhood dental caries and oral-health disparities noted that many preventive initiatives, including school-based programmes, remain fragmented from primary health services and broader community-prevention systems, limiting their potential to reduce caries burden sustainably (27). Even in countries with public insurance schemes, preventive services such as fluoride varnish application and anticipatory guidance receive lower reimbursement rates than treatment services, further disincentivizing prevention at the health-system level. However in case of insurance based oral health policies, referral and utilization of dental services is higher but reports are limited.

Finally, the absence of standardized referral pathways and oral-health screening protocols in pediatric primary care leads to delayed detection of oral diseases. The 2022 WHO report emphasizes that most countries still lack structured oral-health surveillance systems for children, limiting the ability of primary care providers to identify and refer high-risk children early in life. Collectively, these policy and systemic barriers undermine the urgent need for integrated, prevention-centered approaches, including strengthened intersectoral coordination, sustainable financing mechanisms, and workforce planning aligned with child-health needs. Embedding pediatric oral health within broader child-health and universal-coverage agendas remains essential to reducing global inequities and improving long-term outcomes.

## Insurance, finance, and equity dimensions

Financial barriers play a significant role in limiting children's access to oral health care. In many countries, preventive dental services for children are inadequately covered—or not covered at all—by public or private insurance. For example, in the United States, only four in ten Medicaid-enrolled children ages 1–4 received any dental care in 2019, despite mandatory coverage for dental services (28). This gap undermines persistent systemic issues in access and utilization.

High out-of-pocket costs further contribute to delayed treatment and disease progression among lower-income families. Global analyses indicate that household spending on curative dental care can account for 20%–30% of a family's total income in several low- and middle-income countries (29). Such financial strain exacerbates existing inequities in pediatric oral health outcomes. Children from low-income households are 2–3 times more likely to experience ECC and untreated dental disease than their high-income peers (30). Insurance structures that classify dental care as “optional” frequently limit coverage for preventive services, reinforcing these disparities. Also, children with chronic medical conditions and disabilities experience disproportionately greater barriers to oral health care, including higher unmet treatment needs and limited access to clinicians trained in specialized care (31).

Geographic inequities also persist: rural and remote communities often have fewer pediatric dentists per capita and reduced access to timely services (8). Collectively, these economic and systemic barriers contribute to delayed diagnosis, inadequate preventive care, and progression of oral diseases. These findings highlight the need for policy reforms that expand coverage, reduce out-of-pocket burdens, and promote equitable access to oral health services for children.

Opportunities for Integration and Policy Innovation & A Cohesive Roadmap for Pediatric Oral-Health Integration

There is a growing landscape of opportunities to transform health systems, yet the ability to capitalize on them depends on how effectively they are identified, coordinated, and integrated into practice. These opportunities can be organized into a strategic roadmap that supports sustainable implementation and measurable impact across overall health, including oral health. Four domains, in particular, offer high-yield potential:

Advancing interprofessional education and strengthening the health workforce,
Developing and scaling collaborative clinical pathways,
Leveraging digital health innovations and artificial intelligence, and
Promoting equity through financing mechanisms and structural reforms.

Optimizing these domains requires deliberate planning, cross-sector alignment, and robust execution frameworks. When strategically combined, they provide a cohesive platform for improving population health outcomes, reducing disparities, and enhancing system-wide efficiency.

Advancing interprofessional education and strengthening the health workforce

Advancing interprofessional education (IPE) and workforce competencies represents the foundation for advancing pediatric oral-health integration. Multiple evaluations of structured oral-health training programs demonstrate that early exposure to shared competencies, simulation-based clinical activities, and joint pediatric–dental rotations increases trainee preparedness and collaboration readiness (15, 32). These findings build on earlier work showing that structured IPE improves learners’ knowledge, communication, and confidence in preventive counseling and caries-risk assessment (21, 22). As pediatric clinicians play a pivotal role in early detection of ECC, reforms that embed oral-health skills into undergraduate medicine, pediatric residencies, and continuing education can meaningfully reduce variability in practice.

A cohesive policy roadmap therefore begins with sustained educational reform that integrates pediatric oral-health competencies into pre-service and in-service training for medical, dental, nursing, and allied health providers. Embedding shared competencies into accreditation standards, providing mandatory continuing professional development, and supporting longitudinal interprofessional rotations can anchor stable workforce capacity. Evidence from recent curriculum evaluations demonstrates that such structured training improves clinical preparedness, strengthens collaborative communication, and increases the likelihood that clinicians deliver preventive oral-health services in pediatric settings (15, 18). These educational investments establish the foundation for system-wide practice change.

## Developing and scaling collaborative clinical pathways

Opportunities also exist to reinforce collaboration between medical and dental settings by embedding oral-health screening and preventive interventions into routine well-child care. Evidence indicates that simple workflow changes—such as standardized documentation templates, order sets, and point-of-care prompts—can increase pediatric clinicians’ delivery of preventive services, particularly fluoride varnish (33). Multilevel pediatric primary-care interventions that combine provider training, workflow redesign, and structured referral pathways show improved dental attendance and reductions in untreated decay compared with usual care (7). Persisting inconsistencies in preventive delivery continue to highlight the need for aligned incentives, standardized metrics, and interoperable referral frameworks to ensure continuity between medical homes and dental homes (34, 35).

The roadmap therefore requires integrated practice pathways that formalize oral-health screening as a routine element of well-child care and establish reliable referral and follow-up systems between pediatric and dental providers. Policy-driven adoption of standardized screening templates, electronic prompts, fluoride-varnish workflow supports, and referral tracking mechanisms can reduce inconsistencies across clinical settings. Integrating oral-health screening and fluoride-varnish application into routine well-child visits is evidence-based, with research showing that pediatric-delivered preventive care leads to earlier detection of caries and reduced disease burden (36). This approach is reinforced by the Bright Futures preventive-care framework, which embeds oral-health guidance within pediatric supervision to improve continuity of care and promote timely dental referral (35). This model is successful in part because countries with insurance coverage for preventive dental services create financial conditions that support routine oral-health assessments and fluoride-varnish application during well-child visits, facilitating stronger pediatric medical–dental collaboration. Establishing formal partnerships between medical and dental homes further supports continuity of care and early identification of high-risk children.

Another innovative approach could be tasking sharing concept given by WHO. The WHO describes task sharing as a model in which specific clinical responsibilities are redistributed from specialists to trained mid-level or primary-care health workers to strengthen service delivery and expand workforce capacity (37). In alignment with this approach, the Draft Global Oral Health Action Plan 2023–2030 positions task sharing as a key strategy to broaden access to preventive and basic oral-health services—particularly for children—by integrating oral health into primary health-care teams and community-based platforms (38).

## Leveraging digital health innovations and artificial intelligence

Digital-health innovations, including teledentistry, electronic referral systems, and artificial-intelligence tools for caries detection, offer additional opportunities to strengthen system-level integration. Recent reviews report that teledentistry is effective for triage, remote consultation, parent education, and follow-up in pediatric populations, and may enhance access for families in geographically underserved or resource-constrained settings (39, 40). Artificial-intelligence models demonstrate promising accuracy for caries detection across imaging modalities, though authors emphasize the need for rigorous external validation and robust governance structures before clinical deployment (41). When supported by interoperable electronic health records, digital platforms can facilitate referral tracking, population surveillance, and longitudinal coordination across pediatric practices. Digital integration therefore represents the third pillar of the policy roadmap. Scaling teledentistry within pediatric systems can expand access to consultation, triage, and education for underserved families. Interoperable health-information systems linking medical and dental records can generate real-time alerts for high-risk clinical profiles, missed appointments, and targeted outreach opportunities. Artificial-intelligence tools, once externally validated and governed through ethical and technical standards, may enhance diagnostic accuracy for early caries detection. Digital tools show promise for increasing efficiency and reach when accompanied by clear technical guidelines and equity safeguards (39–41). However, it is important to distinguish the importance of conventional risk stratification tools and artificial intelligence dependent digital systems. Digital innovations can enhance conventional approaches, yet in the current stage of their maturity, their role remains largely augmentative—primarily improving referral coordination, policy planning, and precision in preventive targeting.

## Promoting equity through financing mechanisms and structural reforms

Promoting equity through financing and structural policy represents a final domain of opportunity for policymakers. Evidence consistently shows that inequities in access to pediatric dental services are shaped by insurance gaps, cost burdens, availability of pediatric dental providers, and limited incentives for prevention (42, 43). Targeted financing mechanisms—including reimbursement for preventive oral-health services in pediatric visits, explicit coverage of fluoride varnish, and support for community-based and school-based programs—may help reduce financial and structural barriers that disproportionately affect low-income and rural children. These approaches align with global recommendations calling for intersectoral action, community-level initiatives, and universal health coverage that includes essential pediatric oral-health services (8, 26).

Achieving equitable and sustainable improvement in pediatric oral health therefore requires financing reforms that align incentives with prevention and reduce cost-related barriers to care. Ensuring insurance coverage for preventive oral-health services in pediatric primary care, adequately reimbursing providers, and funding community-based programs may help mitigate documented inequities (42, 43). Global guidance indicates that integrating oral health within universal health coverage frameworks is considered important for supporting efforts to reduce ECC and addressing systemic disparities (8). Financing reform ensures that technological and clinical innovations translate into accessible and equitable care for all children.

Thus, to ensure sustainable impact, it is recommended to establish a structured process for regularly reviewing, updating, and enhancing the four strategic domains (interprofessional education, collaborative clinical pathways, digital innovations, and equity-driven reforms). This iterative approach will allow for timely adaptation based on emerging needs, technological advancements, and evolving health system priorities

A concise problem–solution matrix summarizing key barriers to pediatric oral-health integration and the targeted strategies proposed in this study, together with their achievability, equity implications, and anticipated implementation challenges is discussed in Table 2.

**Table 2:** Condensed Problem–Solution–Implementation Matrix for Pediatric Oral-Health Integration.

Problem	Proposed solution	Achievability & evidence	Equity & expected impact	Implementation challenges
Fragmented education and uneven provider competencies.	Unified oral-health curricula; competency-linked accreditation; interprofessional rotations.	High–Moderate; strong post-2020 evidence from IPE and workforce studies.	Strengthens preventive capacity in underserved regions; improves early detection.	Curriculum overload; limited faculty capacity; uneven institutional uptake.
Weak medical–dental integration; unreliable referrals and communication.	Standardized screening templates; EHR prompts; closed-loop referrals; bidirectional communication.	Moderate; moderate evidence from pediatric integration pilots.	More timely referrals for high-risk children; improved continuity of care.	EHR interoperability gaps; workflow resistance; lack of coordination incentives.
Limited digital tools, teledentistry adoption, and AI integration.	Teledentistry expansion; national interoperability standards; validated AI risk tools.	Moderate–Low; emerging evidence but promising digital-health outcomes.	Expands reach to rural/remote populations; earlier disease identification.	Infrastructure cost; digital inequity; data-privacy and AI governance issues.
Structural financing inequities and inconsistent preventive coverage.	Prevention-aligned reimbursement; coverage for varnish/screening; expanded school-based services.	Moderate; strong economic evidence supporting preventive-cost savings.	Lower financial barriers; increased preventive uptake among low-income children.	Policy fragmentation; payer variability; sustainability of community programs.
Policy fragmentation and lack of unified national guidance.	Four-pillar national roadmap with unified metrics and multisector governance.	Moderate; evidence from national oral-health and pediatric policy frameworks.	Reduces geographic disparities; supports scalable, standardized practice.	Divergent political priorities; limited central leadership; funding inconsistency.

## Generalisability

Beyond high-income settings, several non-Western and low- and middle-income countries (LMICs) have begun to explore integrated approaches to pediatric oral health within broader child-health systems. In India, national initiatives such as the National Oral Health Programme emphasize integration of oral health promotion within primary health care and maternal–child health services, particularly through community-based delivery platforms (44). Similarly, Brazil's Unified Health System (SUS) incorporates oral health teams into primary care through the Family Health Strategy, facilitating preventive dental services for children within community-oriented medical care (45, 46).

The integration of oral health into conventional pediatric preventive care from infancy is a critical component. By addressing recommendations for early caries risk evaluation, parental counseling, assessment of fluoride exposure, and the development of a dental home by 12 months of age, it provides a structured approach for integrating medical and dental care. This is a strategy that encourages a policy-based, interdisciplinary approach that is crucial for enhancing child health (47).

These examples illustrate that, even in resource-constrained settings, policy-driven integration of oral health into primary care is feasible and contextually adaptable. However, implementation remains heterogeneous, underscoring the need for workforce training, financing alignment, and governance structures tailored to local health-system capacities.

## Limitations

This review primarily reflects the perspective of dental and oral-health disciplines, with a focus on system-level barriers and integration challenges as described in the dental and public health literature. As a result, the viewpoints of pediatricians, family physicians, nurses, and other primary-care professionals may be underrepresented. While this reflects the current imbalance in published scholarship on pediatric oral-health integration, future interdisciplinary research incorporating medical, nursing, and caregiver perspectives will be essential to develop more comprehensive and operationally feasible integration models.

## Conclusion

Pediatric oral health remains a critical component of overall child well-being, yet it is often siloed from broader primary care services. Evidence highlights persistent gaps in professional training, collaboration between medical and dental providers, and system-level integration, which contribute to inconsistent preventive care and inequities in access. Recognizing oral health as integral to general health reinforces the need for policies and interventions that are contextually adaptable and responsive to local health-system capacities, including examples from both high-income and low- and middle-income countries.

Integrating pediatric oral health into primary care, supported by interprofessional education, collaborative clinical pathways, and digital innovations, has the potential to improve care coordination and access. Equity-driven reforms are essential to address disparities and ensure that children in underserved populations benefit from preventive and coordinated services. While existing evidence demonstrates promising approaches, continued interdisciplinary research and system-level evaluation are required to refine best practices and tailor interventions to diverse healthcare contexts.

To ensure sustainable impact, it is recommended to establish a structured process for regularly reviewing, updating, and enhancing the four strategic domains—interprofessional education, collaborative clinical pathways, digital innovations, and equity-driven reforms. This iterative approach allows timely adaptation based on emerging needs, technological advancements, and evolving health system priorities, thereby supporting long-term integration of pediatric oral health within child health services globally.
